# Morphometric analysis of the axillary nerve in relation to the deltopectoral and deltoid-splitting approaches: implications for surgical safe zones

**DOI:** 10.1007/s00276-026-04018-2

**Published:** 2026-10-09

**Authors:** Bronwyn Lottino, Erik Hohmann, Jade Naicker, Nkhensani Mogale

**Affiliations:** 1 Department of Anatomy, School of Medicine, Faculty of Health Sciences, University of Pretoria, Dr Savage Rd, Prinshof 349-Jr, Pretoria, 0084 South Africa; 2 Department of Orthopaedic Surgery and Sports Medicine, Valiant Clinic/Houston Methodist Group, Dubai, United Arab Emirates

**Keywords:** Axillary nerve, Deltopectoral approach, Shoulder surgical anatomy, Surgical safe zones, Sex-specific shoulder morphometry

## Abstract

**Purpose:**

The deltopectoral splitting approach is commonly used in shoulder surgery; however, injury to the axillary nerve remains a major concern due to potential motor and sensory deficits. This study aimed to define the anatomical relationships of the axillary nerve to surrounding structures in order to optimize surgical access and minimize the risk of iatrogenic injury.

**Methods:**

Thirty formalin-fixed cadavers (15 males, 15 females) were dissected. Quantitative measurements were obtained to determine the position of the axillary nerve relative to the deltopectoral interval and deltoid muscle. Descriptive statistics, reported as means ± standard deviations (SD), were calculated, and independent t-tests were used to evaluate differences based on sex and laterality.

**Results:**

Sex-specific differences were observed in axillary nerve positioning. The superior safe zone, measured from the acromion to the anterior branch of the axillary nerve, averaged 65.31 ± 10.82 mm in females and 71.18 ± 9.72 mm in males. The distance from the anterior border to the anterior branch was 48.72 ± 10.89 mm in both sexes. In contrast, the posterior branch measured 21.45 ± 9.25 mm in females and 33.03 ± 22.74 mm in males. No significant differences were observed between left and right shoulders (*p* ≥ 0.11).

**Conclusion:**

The deltopectoral approach provides reliable access to the glenohumeral joint while minimizing axillary nerve injury when anatomical variations are considered. These findings underscore the importance of sex-specific morphometric considerations and support the integration of population-specific anatomical data into surgical planning to enhance safety and optimize clinical outcomes.

## Introduction

Developing safe and effective nerve-sparing surgical techniques remains a fundamental challenge in modern medical practice. The axillary nerve plays a critical role in both motor and sensory innervation of the shoulder girdle. This nerve supplies the deltoid and teres minor muscles, which are crucial for arm abduction and external rotation [8]. Additionally, the axillary nerve gives rise to the superior lateral cutaneous nerve of the arm, providing sensory innervation to the skin overlying the deltoid region [5]. The axillary nerve traverses the quadrangular space before branching into its motor and sensory components, thereby facilitating movements essential for daily activities and athletic performance [5, 26]. Lottino et al. demonstrated substantial variability in axillary nerve branching patterns, by classifying the nerve into four branching types, with subtypes [16]. This highlights the need to recognize these anatomical variations to improve surgical and anaesthetic planning and reduce the risk of nerve injury.

Axillary nerve injuries may result from direct trauma, shoulder dislocation, proximal humeral fractures, or iatrogenic causes during surgical intervention. The severity of the injury ranges from transient neuropraxia to more severe forms such as axonotmesis and neurotmesis [14], potentially leading to significant motor deficits, particularly impaired arm abduction and external rotation [2]. Although surgical approaches such as the deltoid-splitting approach are often employed in rotator cuff repairs and shoulder arthroplasty with the aim of minimizing nerve damage, anatomical variations in the course of the axillary nerve continue to pose a substantial risk [1]. Despite extensive study, there remains no consensus regarding a consistent and standardized ‘safe zone’ to reliably prevent axillary nerve injury. Furthermore, surgical outcomes may heavily rely on the diverse variations in branching types, which directly contribute to a higher risk of iatrogenic injury due to an unawareness of these specific axillary nerve branching patterns [16].

The deltopectoral approach is widely employed for open reduction and internal fixation (ORIF) of proximal humeral fractures. While it may be associated with soft tissue trauma and potential injury to the anterior circumflex humeral artery [27, 19], it provides excellent surgical exposure and carries a comparatively lower risk of axillary nerve injury, making it particularly suitable for shoulder arthroplasty [24, 9]. In contrast, the anterolateral approach offers direct access to the lateral humerus with minimal muscle retraction; however, it carries a greater risk of nerve injury due to the required dissection [17]. The lateral or deltoid splitting approach is less invasive and offers better access to the glenoid cavity and tuberosities; however, the axillary nerve remains vulnerable, and this technique has been associated with less favourable clinical outcomes compared to the deltopectoral approach [20, 10]. Furthermore, the lateral or deltoid splitting approach is less invasive as the incision required to access the glenoid cavity and tuberosities is smaller, allowing for direct exposure of the landmarks [21]. The posterior approach, while preserving the rotator cuff and offering excellent visualization, is associated with longer recovery times and continued risk of nerve injury [6, 7]. In the clinical literature, these techniques are precisely classified as the anterior deltoid-splitting [11], mid-deltoid (lateral) splitting [23], and posterior deltoid-splitting approaches [13], each presenting unique structural risks to the branches of the axillary nerve.

As such, this study aimed to define reliable safe zones to minimize axillary neve injury during surgical access to the glenohumeral joint. Particular emphasis is placed on evaluating clinical relevance and efficacy of the deltopectoral splitting approach in preserving the integrity of the nerve. Through detailed cadaveric dissection and morphometric analysis, this investigation seeks to enhance surgical precision and reduce the risk of postoperative complications.

## Materials and methods

### Materials

To evaluate the risk of axillary nerve injury associated with the deltopectoral approach, a quantitative cadaveric study was conducted. Thirty formalin-embalmed cadavers (*n* = 30), comprising of 15 females and 15 males, were obtained from the Department of Anatomy, University of Pretoria. Bilateral dissections were performed, yielding a total of 60 shoulders for analysis. Ethical approval was granted by the Faculty of Health Sciences Research Ethics Committee, University of Pretoria (Ethics number: 455/2024), and the research was conducted in accordance with the National Health Act no. 61 of 2003. The study was performed in accordance with the ethical standards as laid down in the 1964 Declaration of Helsinki and its later amendments or comparable ethical standards.

The combined sample demonstrated the following mean demographic characteristics: age 71.43 years (SD ± 14.11), weight 64.49 kg (SD ± 17.43), height 1.65 m (SD ± 0.08), and body mass index (BMI) 24.06 kg/m² (SD ± 5.99). Female demographic characteristics were as follows: age 76.40 ± 12.11 years, weight 62.68 ± 16.67 kg, height 1.61 ± 0.06 m, and BMI 24.94 ± 5.93 kg/m^2^. Male demographic characteristics were as follows: age 66.47 ± 13.98 years, weight 68.95 ± 17.67 kg, height 1.69 ± 0.07 m, and BMI 24.62 ± 6.29 kg/m^2^. All morphometric measurements were obtained using a calibrated sliding calliper with an accuracy of 0.01 mm.

Inclusion criteria required cadavers to have intact shoulder regions with preserved axillary nerves. Specimens with evidence of prior surgical intervention, pathological changes, trauma, or anatomical abnormalities affecting the shoulder region were excluded. This ensured the examination of anatomically intact specimens, thereby allowing accurate assessment of the axillary nerve and its relationship to the deltopectoral approach without confounding variables.

### Methods

To facilitate safe exposure of the axillary nerve during dissection procedures, palpation of specific anatomical landmarks was employed. The anteromedial aspect of the coracoid process was palpated and marked to approximate the region where the axillary nerve courses before entering the quadrangular space. Additionally, the posterolateral aspect of the acromion was palpated and marked to indicate the area where the nerve enters the deltoid muscle. 

Dissections were performed in accordance with the guidelines outlined in Grant’s Dissector [4]. A longitudinal skin incision was made from the acromion to the deltoid tuberosity and reflected laterally to expose the underlying deltoid muscle. The deltoid was then carefully detached from its origins: the posterior fibres from the inferior border of the spine of the scapula, the middle fibres from the lateral aspect of the acromion, and the anterior fibres from the lateral third of the clavicle. The muscle was subsequently reflected toward its insertion to allow full visualization of the axillary nerve. 

**Fig. 1:**
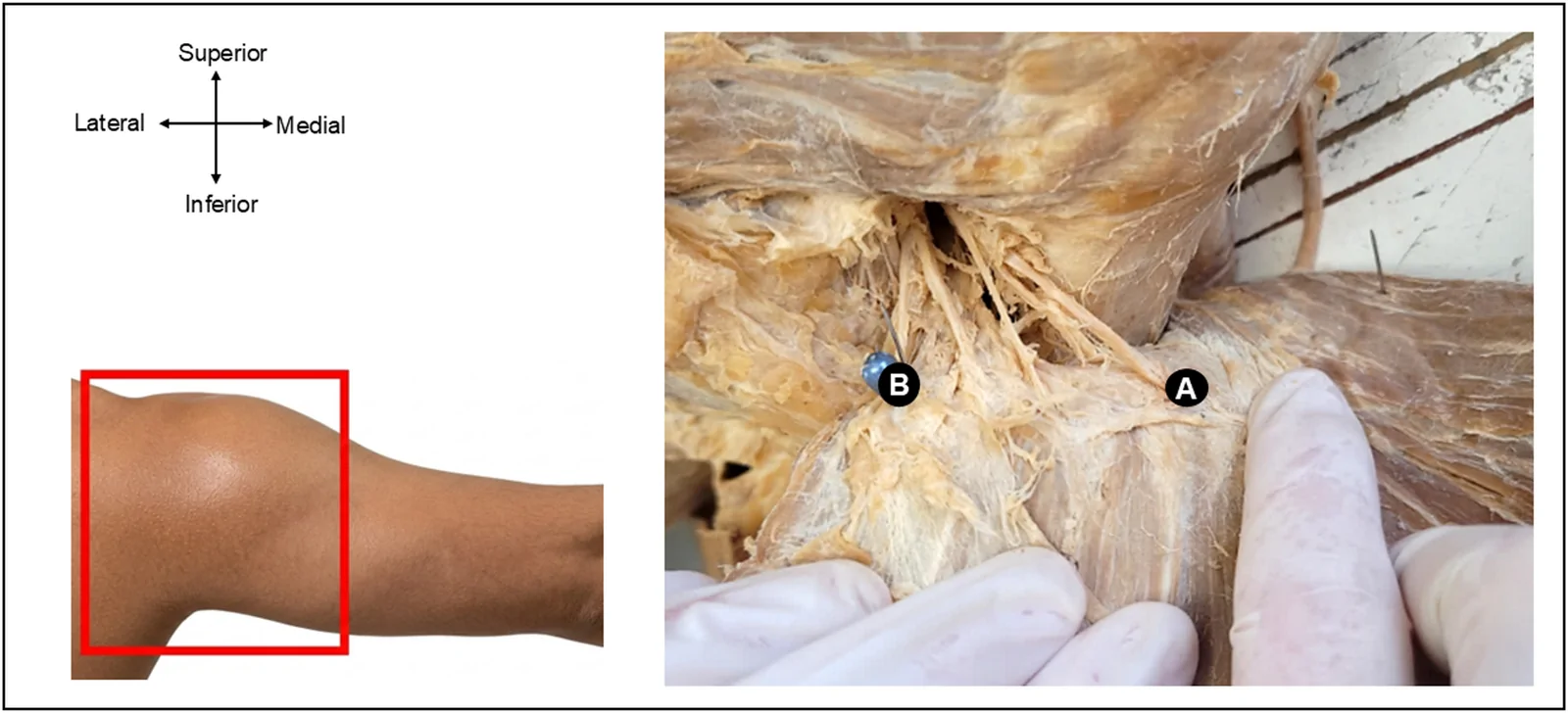
Posterior view of the shoulder with the deltoid muscle reflected inferiorly. **A** – anterior branch of the axillary nerve, **B** – posterior branch of the axillary nerve

**Fig. 2:**
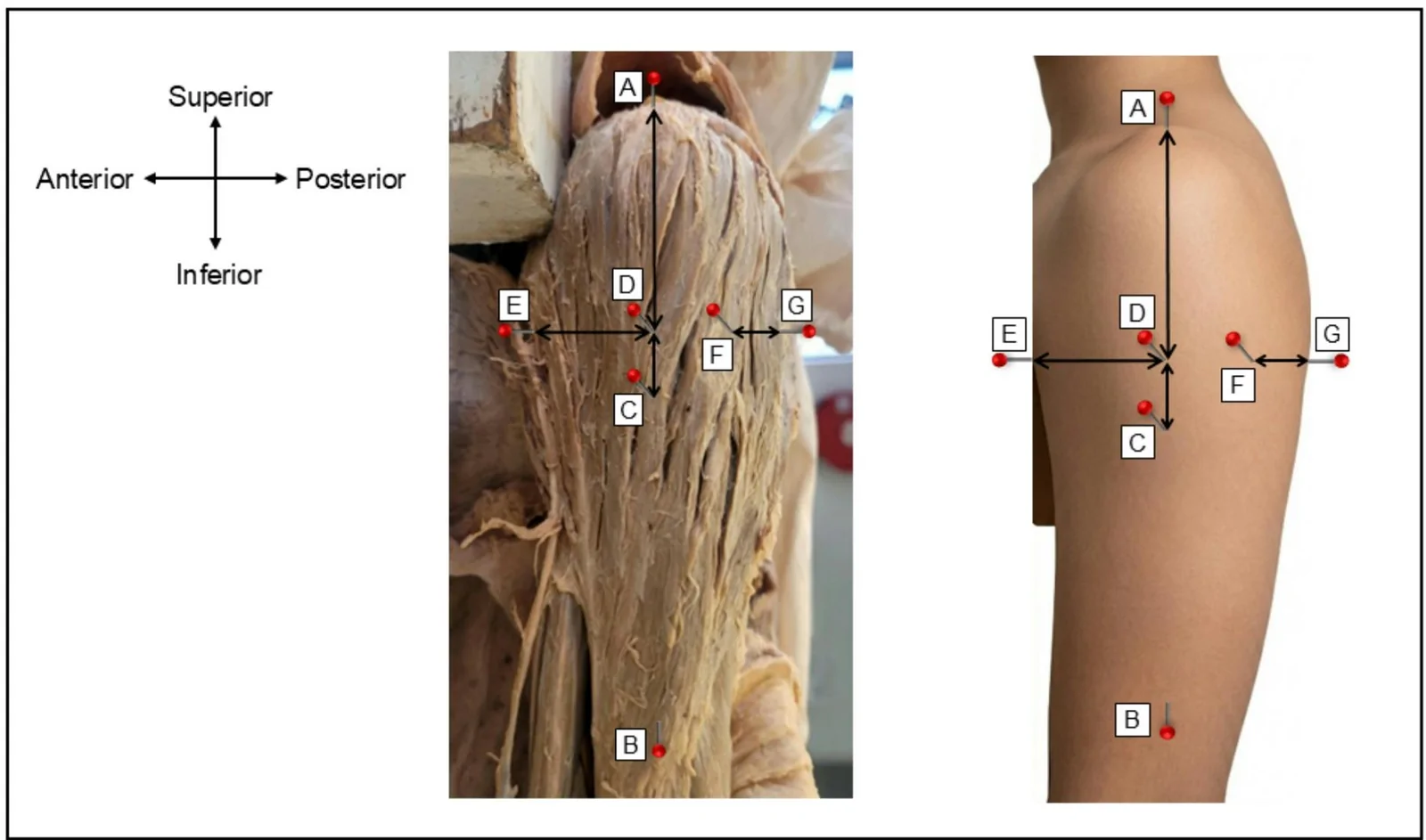
Lateral view of the shoulder with the deltoid muscle visible, illustrating the anatomical landmarks used for measurements. **A** – the most lateral aspect of the acromion, **B** – the most inferior point of the deltoid tuberosity, **C** – midpoint between point A and B along the same vertical plane, **D** – location of the anterior branch of the axillary nerve, **E** – the most anterior aspect of the deltoid muscle, **F** – location of the posterior branch of the axillary nerve along the same plane, **G** – the most posterior aspect of the deltoid muscle along the same plane

Cadavers were positioned in the prone position, and the deltoid muscle was reflected inferiorly to expose the axillary nerve. The anterior and posterior branches of the nerve were carefully pinned through the muscle for measurement (Figure 1, A–B). Following this, the deltoid muscle was returned to its original position and secured, and the following surface anatomical landmarks were palpated:

- The most lateral aspect of the acromion (Fig. 2, A).
- The posterolateral aspect of the acromion.
- The posterolateral aspect of the acromion was pinned to mark the approximate location of the axillary nerve on the surface.

**Fig. 3:**
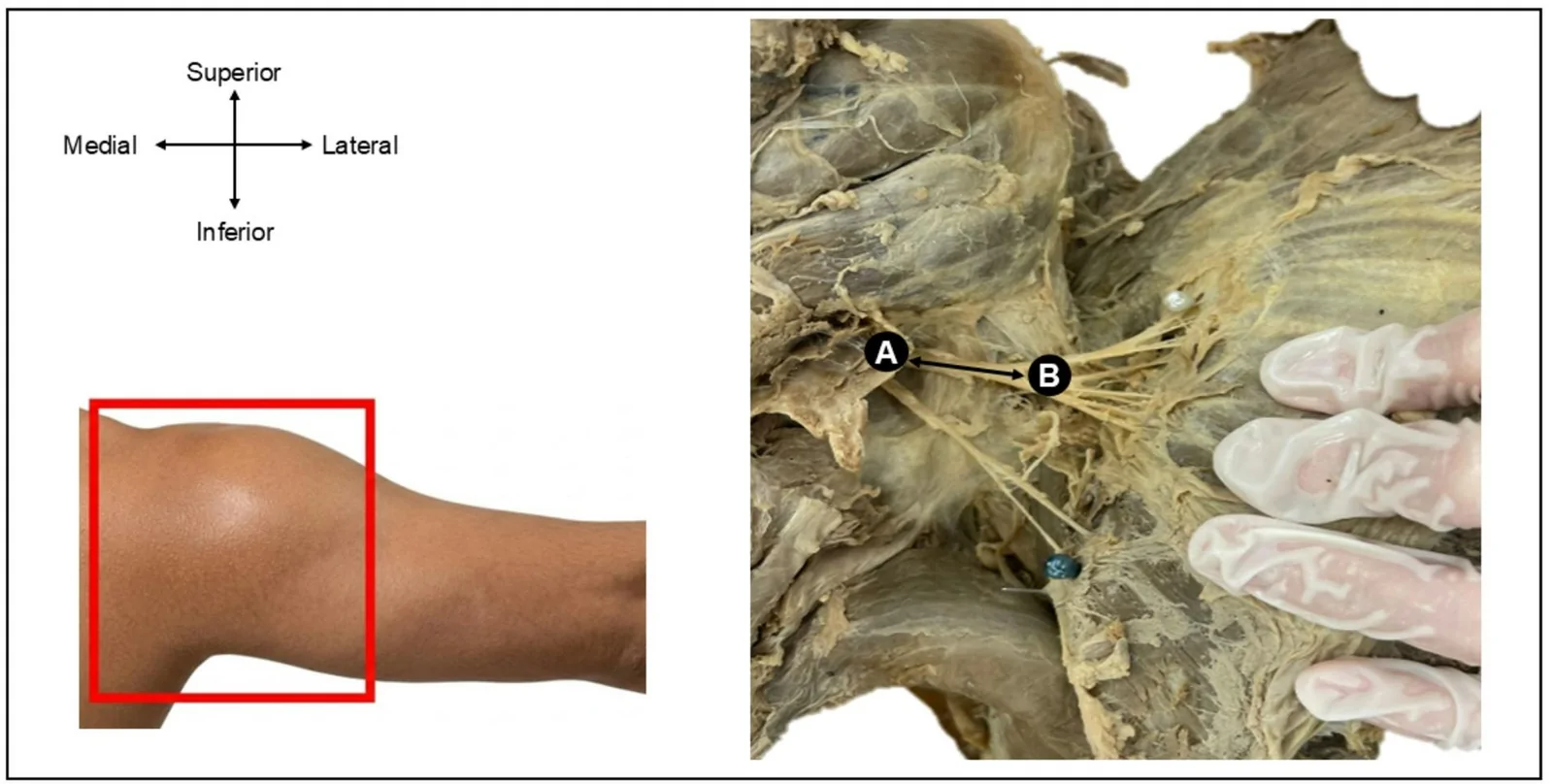
Posterior view of the shoulder with the deltoid muscle reflected inferolaterally, illustrating the distance from the axillary nerve as it exits the quadrangular space (**A**) to its branching into the anterior and middle branches (**B**)

**Fig. 4:**
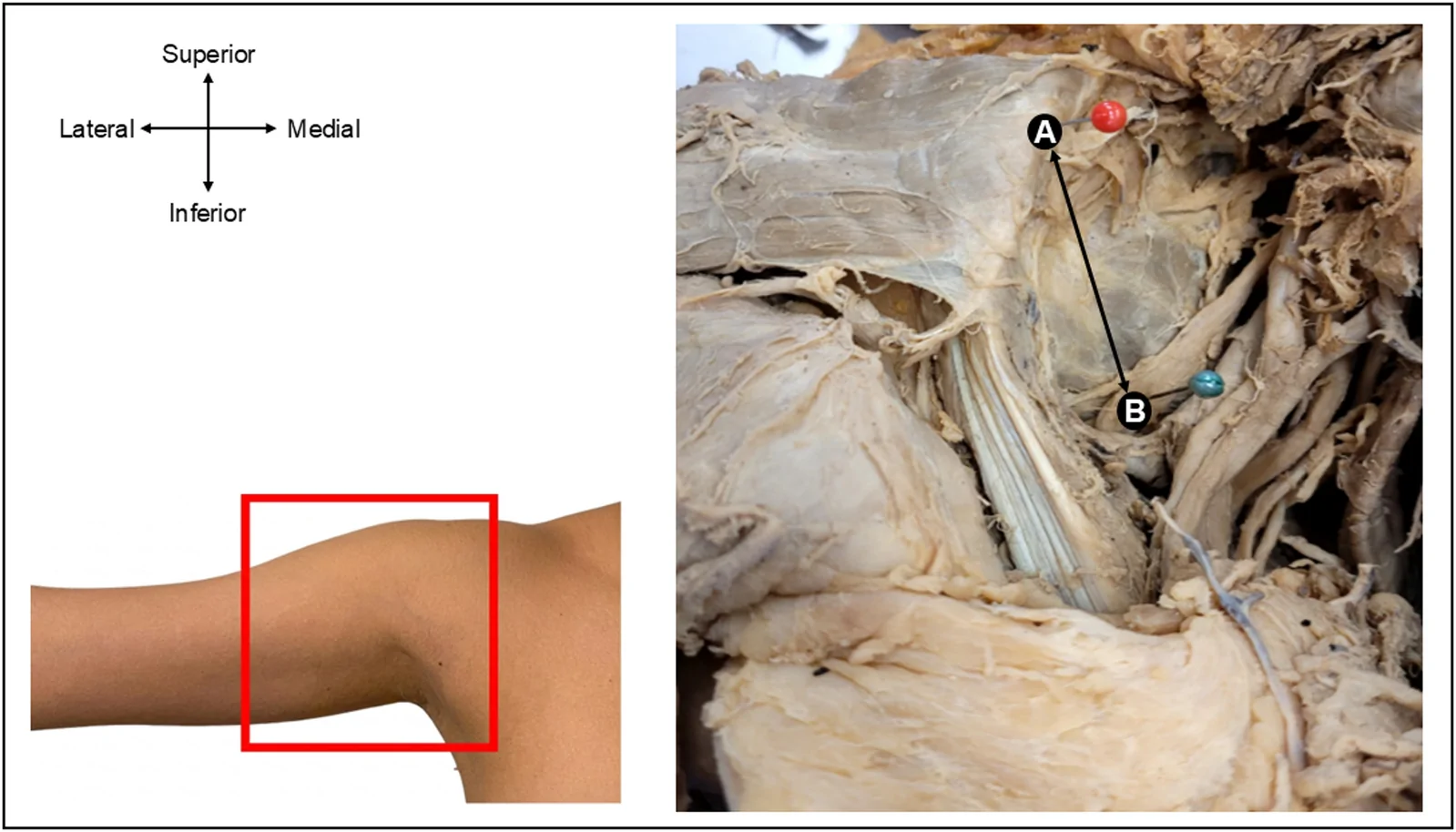
Anterior view of the shoulder showing the axillary nerve coursing through the quadrangular space. **A** – anteromedial tip of the coracoid process, **B** – axillary nerve coursing through the quadrangular space

The following anatomical measurements were recorded to map the course and branching of the axillary nerve:

- The distance from the most lateral point of the acromion (Fig. 2, A) to the most inferior point of the deltoid tuberosity (Fig. 2, B). The midpoint of this vertical plane was also measured (Fig. 2, C).
- The distance from the anterior branch of the axillary nerve (Fig. 2, D) to the midpoint of the vertical deltoid distance (Fig. 2, C).
- The linear distance from the lateral point of the acromion to the anterior branch of the nerve along the same plane (Fig. 2, A–D).
- The horizontal distance from the anterior branch of the axillary nerve to the anterior border of the deltoid muscle (Fig. 2, D–E).
- The horizontal distance of the posterior branch to the posterior border of the deltoid muscle (Fig. 2, F–G).
- The distance from the quadrangular space to the point where the nerve began branching (Fig. 3).
- With the cadaver repositioned supine, the distance from the axillary nerve’s entry point into the quadrangular space, to the anteromedial tip of the coracoid process (Fig. 4, A–B).

Descriptive statistics, including the mean, range, standard deviation, and 95% confidence intervals, were calculated for all measured variables. Pearson’s correlation coefficient was used to evaluate the relationship between cadaver demographic data and measurements. Several statistical analyses were conducted to ensure the validity of the findings: paired t-tests were used to determine significant differences between left and right shoulder measurements; unpaired* t*-tests were conducted to compare measurements between male and female cadavers. Additionally, intraclass correlation coefficients were calculated to assess both intra-observer and inter-observer reliability, thereby evaluating the accuracy of the results and reproducibility to the measurement methods.

## Results

The analysis of shoulder measurements yielded consistent and clinically relevant findings. Paired t-tests comparing left and right shoulders demonstrated no statistically significant differences (*p* ≥ 0.11), indicating symmetry between sides. In contrast, unpaired t-tests comparing male and female measurements revealed statistically significant differences in nearly all parameters (*p* ≤ 0.04), with exceptions including the horizontal distance from the anterior branch to the anterior border of the deltoid at the acromion (*p* = 0.30) and the distance from the quadrangular space to the nerve’s branching point (*p* = 0.26). These results highlight marked sexual dimorphism in axillary nerve positioning.

**Fig. 5:**
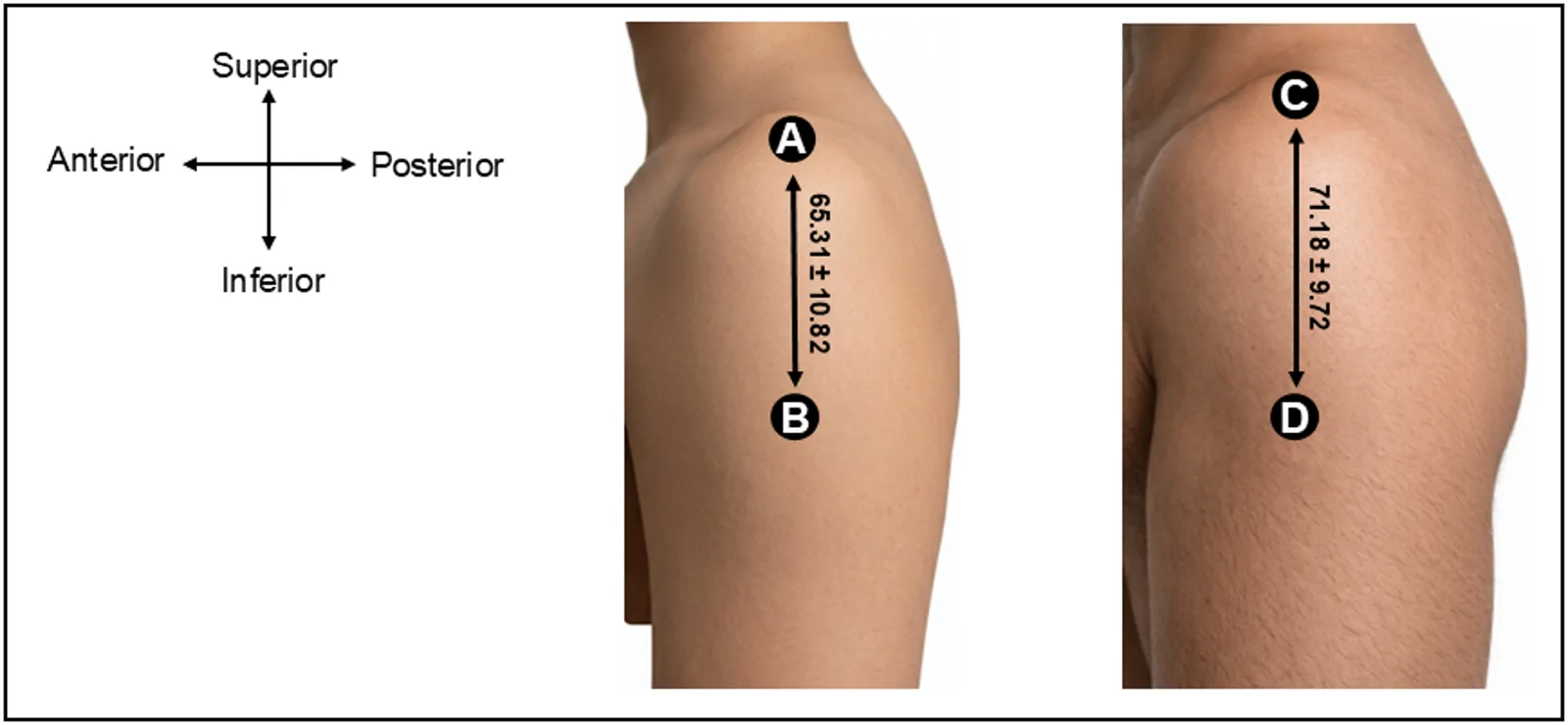
Lateral view of the shoulder indicating the safe zones for males and females. **A** – lateral aspect of acromion (females), **B** – anterior branch of the axillary nerve (females), **C** – lateral aspect of the acromion (males), **D** – anterior branch of axillary nerve (males)

The distance from the acromion to the anterior branch of the axillary nerve defined the superior safe zone. Females demonstrated a mean distance of 65.31 ± 10.82 mm, while males averaged 71.18 ± 9.72 mm (Fig. 5).

The relationship between the axillary nerve and the deltoid muscle are summarized in Table 1. The anterior branch was consistently positioned 48.72 ± 10.89 mm from the anterior border of the deltoid in both sexes. In contrast, the posterior branch exhibited sex-specific differences, measuring 21.45 ± 9.25 mm from the posterior deltoid margin in females and 33.03 ± 22.74 mm in males.

**Table 1:** Topographical relationships of the axillary nerve branches to the deltoid muscle

Measurement	Female mean ± SD (mm)	Male mean ± SD (mm)	Clinical note
Horizontal distance from the anterior branch of the axillary nerve to the anterior border of the deltoid muscle	48.72 ± 10.89	48.72 ± 10.89	Consistent surgical clearance for the deltopectoral approach
Horizontal distance from the posterior branch of the axillary nerve to the posterior border of the deltoid muscle	21.45 ± 9.25	33.03 ± 22.74	Greater posterior surgical clearance was observed in males
Distance from the point where the axillary nerve enters the quadrangular space to the anteromedial tip of the coracoid process	56.23 ± 6.32	60.54 ± 5.44	This landmark is important for avoiding nerve injury during deep dissection

Measurement reliability was assessed by comparing inter-observer and intra-observer values with the original dataset. Overall, the results demonstrated excellent reliability, with inter-observer correlation coefficients exceeding 0.98 and intra-observer coefficients above 0.96, indicating high repeatability and consistency of the measurements. A few exceptions were noted: the horizontal distance from the anterior branch to the anterior border of the deltoid at the acromion on the left side showed only a moderate correlation of 0.59, suggesting reduced reliability for this measurement. Similarly, the horizontal distance from the posterior branch to the posterior border of the deltoid on the left side exhibited a moderate correlation coefficient of 0.61 [12].

## Discussion

Morphometric analysis revealed that the distance from the acromion to the anterior branch of the axillary nerve averaged 65.31mm ± 10.82 mm in females and 71.18 cm ± 9.72mm in males. These data delineate a population-specific 'superior safe zone' that is notably more constricted than the traditionally cited 50–80 mm parameters [25]. However, the conventional '5 cm rule' may offer sufficient margin of safety in South African cohorts, although among female patients, the nerve exhibits a more inferiorly displaced course. An extensive review of literature identified a lack of evidence regarding these anatomical boundaries. Consequently, the present study represents the first formal establishment of such safe zones in this specific cohort using a formalin-fixed cadaveric model.

### The deltopectoral approach

Topographical analysis revealed that the anterior branch of the axillary nerve maintains a highly consistent distance of 48.72 ± 10.89 mm from the anterior border of the deltoid muscle across both sexes. Given that the standard deltopectoral approach exploits the intermuscular interval immediately adjacent to this anatomical boundary, these morphometric data underscore the relative safety of this pathway. It provides an expansive anatomical clearance zone that minimizes the risk of direct nerve trauma when contrasted with transdeltoid splitting techniques. However, a critical clinical nuance emerges regarding sexual dimorphism. While male cohorts exhibit a comparatively larger anatomical window, the significantly more constricted safe zones identified in female specimens dictate heightened intraoperative vigilance. This sex-specific vulnerability necessitates extreme precision during high-risk maneuvers, such as the placement of deep subacromial retractors, to mitigate the risk of iatrogenic nerve compromise.

### The anterolateral (mid-deltoid/lateral splitting) approach

The anterolateral approach facilitates direct exposure of the lateral aspect of the humerus and the glenohumeral joint while minimizing the necessity for extensive soft-tissue retraction. However, its clinical utility is constrained by an elevated risk of neurovascular injury secondary to the mandatory vertical transdeltoid dissection. The safety of this direct lateral splitting technique is inherently dependent on the preservation of the superior safe zone, topographically defined as the vertical distance from the lateral border of the acromion to the horizontally coursing anterior branch of the axillary nerve. The morphometric findings of this study (65.31 ± 10.82 mm in females and 71.18 ± 9.72 mm in males) indicate that while the traditional "5 cm rule" remains a statistically viable guidelines for surgical clearance within this cohort, female cohorts exhibit a significantly more superiorly displaced neural trajectory. This pronounced sex-specific restriction of the superior safe zone implies that the permissible threshold for vertical muscle splitting must be stringently curtailed in female patients to mitigate the risk of catastrophic iatrogenic transection of the primary nerve bundle.

### The anterior deltoid-splitting approach

Utilization of the anterior deltoid-splitting approach demands rigorous compliance with established horizontal anatomical parameters to avoid neurological compromise. Because the anterior branch of the axillary nerve follows a transverse trajectory, maintaining a uniform morphometric clearance of 48.72 ± 10.89 mm from the anterior border of the deltoid muscle across both sexes, any vertical division of these anterior muscular fibers must be stringently restricted within this medial anatomical boundary. Encroachment beyond this structural threshold subjects the primary motor branch to immediate mechanical deformation or transaction. Clinically, such iatrogenic disruption precipitates denervation, leading to irreversible atrophy of the anterior deltoid belly and a profound, permanent deficit in glenohumeral abduction torque.

### The posterior deltoid-splitting approach

The posterior deltoid-splitting approach introduces a highly critical operative constraint dictated by pronounced sexual dimorphism within the target cohort. Morphometric analysis located the posterior branch of the axillary nerve at a mean distance of 21.45 ± 9.25 mm from the posterior deltoid margin in female specimens, compared to a significantly more expansive 33.03 ± 22.74 mm in males. The topographical variations quantified in this investigation likely represent a macroscopic correlate of overall physical stature, regional deltoid muscle volume, and longitudinal humeral morphology. Given that the posterior safe zone is markedly reduced in females, measuring just over 21 mm, the use of a posterior vertical split or the placement of deep subacromial retractors may place the axillary nerve at increased risk of mechanical injury. Consequently, the margin for error is drastically reduced in female patients, predisposing them to a higher incidence of iatrogenic nerve trauma and subsequent neurological deficits compared to their male counterparts.

The topographical variations observed in this study likely represent a morphometric correlate of overall physical stature [18], deltoid muscle volume [22], and humeral length. These findings suggest that while the deltopectoral approach offers a relatively larger anatomical window in male patients, the significantly more constricted safe zones in females necessitate heightened intraoperative vigilance. This sex-specific vulnerability is particularly critical during high-risk surgical manoeuvres, such as the placement of subacromial retractors or the positioning of proximal humeral implants.

The clinical awareness of these narrower margins is underscored by the debilitating functional consequences of iatrogenic axillary nerve injury. Denervation of the anterior branch typically results in irreversible deltoid muscle atrophy and a profound deficit in shoulder abduction. Given that such injuries lead to permanent functional impairment and significantly diminished patient quality of life, these population-specific safe zones provide essential parameters for mitigating surgical risk.

**Fig. 6:**
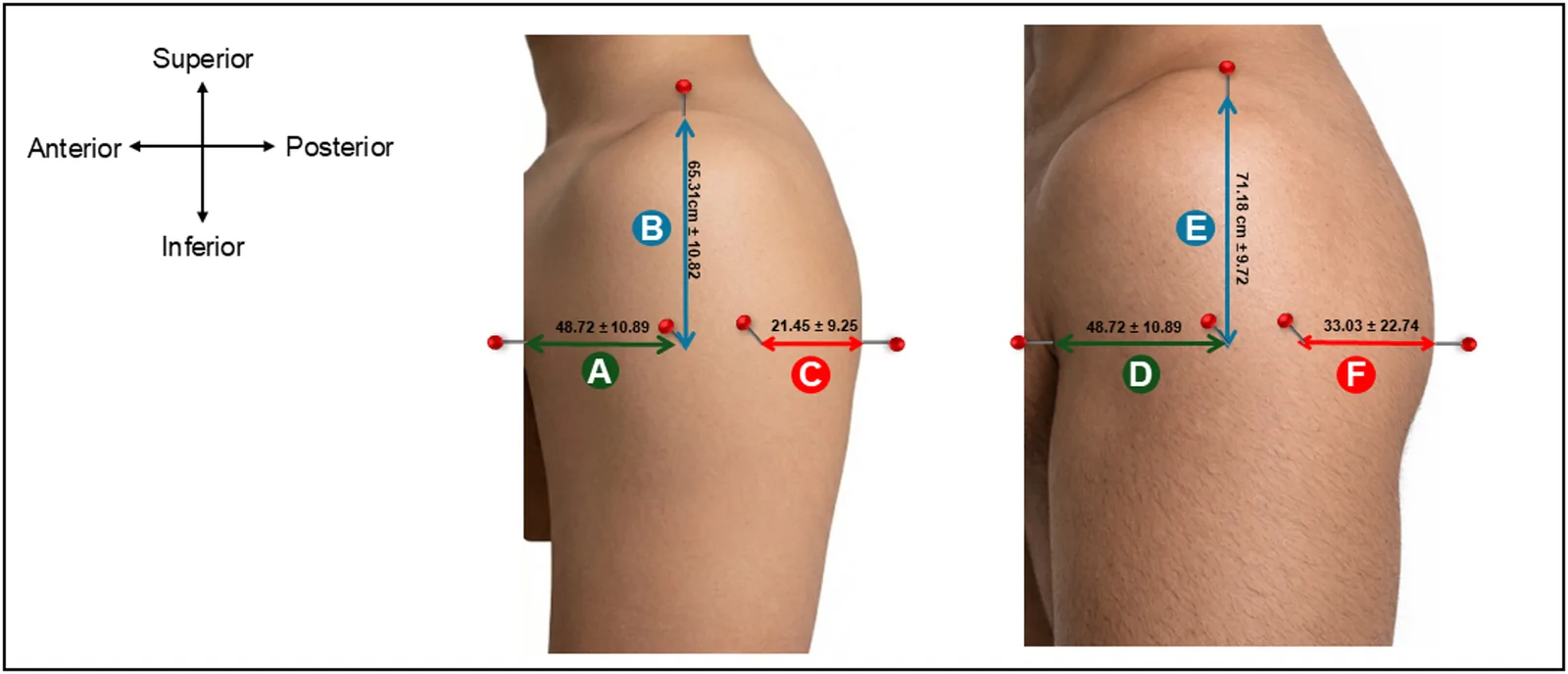
Lateral view of the shoulder showing: **A** – the anterior surgical clearance safe zone in females (green), **B** – the super surgical clearance safe zone in females (blue), **C** – the posterior surgical clearance safe zone in females (red), **D** – the anterior surgical clearance safe zone in males (green), **E** – the super surgical clearance safe zone in males (blue), **F** – the posterior surgical clearance safe zone in males (red)

Figure 6 below illustrates these established safe zones, providing a visual guide for the superior, anterior, and posterior boundaries identified in this South African cohort.

The importance of adhering to these population-specific safe zones is underscored by the significant morbidity associated with iatrogenic injury to the axillary nerve. While some literature suggests that perioperative neurologic symptoms may be transient, evidence from longitudinal follow-ups of the Latarjet procedure indicates that axillary nerve involvement frequently results in protracted morbidity that fails to resolve years postoperatively [22]. Specifically, patients with axillary nerve dysfunction have been shown to suffer from persistent sensory disturbances, with a significant cohort experiencing residual motor weakness that remains unresolved at final clinical follow-ups [22].

Table 2 summarizes reported incidences of axillary nerve injury during the deltopectoral approach, ranging from 0 to 33%. Direct comparison of nerve–deltoid distances with the literature is not possible, as prior studies reported only incidence rates rather than quantitative morphometric data. Consequently, this study provides novel, clinically relevant morphometric measurements for a South African population that can inform surgical strategy and improve procedural safety.

**Table 2:** Reported incidence of axillary nerve injury during deltopectoral shoulder approaches in previous studies

Study	Sample size	Population	Incidence of axillary nerve damage	Other structures damaged
Stecco et al. [25]	38	Italian	1.0–4.3%	Soft tissue
Choi et al. [3]	869	South-Korean	0.3–0.9%	Median nerve, Musculocutaneous nerve
LiBrizzi et al. [15]	77	American	0–33.0%	Cephalic vein

These findings have direct clinical implications. Surgeons can use the sex-specific safe zones to navigate surgical incisions and dissections particularly during the deltopectoral splitting approach and the anterolateral approach, reducing the likelihood of iatrogenic injury. Additionally, these data can inform preoperative imaging protocols, surgical simulation training, and intraoperative navigation, particularly in populations for which existing morphometric data are limited.

Future studies should aim to correlate these morphometric measurements with actual surgical outcomes, including nerve injury incidence in posterior approaches, and explore potential differences across other populations. The integration of high-resolution ultrasound and MRI during preoperative planning allows for the translation of these morphometric averages into patient-specific anatomy. Utilizing imaging to map the individual depth and superior-inferior position of the axillary nerve relative to the acromion could further enhance surgical simulation training and intraoperative navigation, particularly in populations where existing anatomical data are limited. Image-guided nerve mapping, may therefore provide an additional strategy for improving surgical safety. However, the available literature is heterogeneous and lacks sufficient comparable clinical data to reliably pool injury rates between image-guided and conventional landmark-guided techniques. Future clinical studies are needed to determine whether image-guided identification of the axillary nerve can provide a meaningful advantage over conventional approaches to further refine anatomical guidance for safer shoulder surgery.

The proposed safe zones provide a basis for subsequent validation studies using fresh-frozen specimens and imaging modalities to determine the clinical applicability of these measurements and whether image-guided identification of the axillary nerve can further improve accuracy and utility. Such studies should also help establish patient-specific anatomical reference points that may complement conventional surgical landmarks.

The primary limitation of this study is the limited number of documented axillary nerve injuries associated with different surgical approaches in the available literature. This scarcity of clinical outcome data limits the ability to accurately assess the relative effectiveness of different techniques in preserving the nerve and precludes robust comparative analyses. Contributing factors may include underreporting in clinical practice, variation in surgical techniques, and inconsistencies in the diagnostic criteria used to identify nerve injury. In addition to these limitations related to the available clinical evidence, several methodological limitations in the present study should be acknowledged. First, cadaveric measurements may differ slightly from in vivo anatomy due to post-mortem changes and tissue shrinkage. Second, although the sample size was sufficient for morphometric analysis, it may not fully capture the full spectrum of anatomical variation in the broader population. Finally, despite high inter- and intra-observer reliability, the measurements may still be influenced by variations in landmark identification and specimen positioning.

Future research should focus on collecting larger, more comprehensive datasets across diverse surgical approaches to better understand the associated risks and develop evidence-based guidelines. Addressing these limitations is essential to enhance patient safety, optimize surgical outcomes, and advance knowledge in axillary nerve preservation.

## Conclusion

This study demonstrates that the deltopectoral splitting approach provides the greatest anatomical clearance for the axillary nerve, supporting its use for glenohumeral joint access while minimizing iatrogenic injury. Based on the morphometric data from this South African cohort, incisions or deep dissections should not extend more than 65 mm inferior to the acromion in females and 71 mm in males, while remaining at least 48 mm from the anterior deltoid border to protect the anterior branch. The posterior safe zone was significantly narrower in females (21.45 ± 9.25 mm) than in males (33.03 ± 22.74 mm). No significant differences were observed between left and right shoulders, whereas significant sex-related differences were identified, highlighting the importance of sexual dimorphism and population-specific anatomy when defining safe zones. These findings can inform preoperative planning and intraoperative dissection to reduce the risk of axillary nerve injury and provide a basis for future clinical validation.

## Data Availability

The datasets generated and/or analyzed during the current study are available from the corresponding author on reasonable request.
